# Hybrid RNA sequencing of broad bean wilt virus 2 from faba beans

**DOI:** 10.1128/spectrum.02663-23

**Published:** 2023-10-12

**Authors:** Solomon Maina, Sally L. Norton, Brendan C. Rodoni

**Affiliations:** 1 NSW Department of Primary Industries, Biosecurity & Food Safety, Elizabeth Macarthur Agricultural Institute, Woodbridge Road, Menangle, NSW, Australia; 2 Australian Grains Genebank, Agriculture Victoria, Horsham, Victoria, Australia; 3 Microbial Sciences, Pests & Diseases, Agriculture Victoria, AgriBio, Ring Road, Bundoora, Victoria, Australia; 4 School of Applied Systems Biology (SASB), La Trobe University, Bundoora, Victoria, Australia; USDA - San Joaquin Valley Agricultural Sciences Center, Parlier, California, USA

**Keywords:** high-throughput sequencing, plant viruses, broad bean wilt virus 2

## Abstract

High-throughput sequencing (HTS) is an important tool for plant virus detection and discovery. HTS technologies such as Nanopore sequencing has been rapidly developing in recent years, and offers new possibilities for fast routine diagnostic applications. This study compared MiSeq (Illumina) RNA-Seq with the MinION sequencer (Oxford Nanopore Technologies) (ONT) direct-RNA-Seq methods to detect and sequence the broad bean wilt virus 2 (BBWV2) genome. The Illumina BBWV2 RNA1 and RNA2 genome segments were intact and matched with ONT with 99.1% and 98.8% nucleotide identity (nt) match, respectively. The RNA1 genome derived from ONT had deletions within the nucleoside-triphosphate binding and protease factor open-reading frames, and the upstream of five untranslated regions. However, its RNA2 was intact, and no sequencing errors were observed. The ONT and Illumina RNA1 and RNA 2 BBWV2 genome segments clustered together phylogenetically along with other none-*Fabaceae* species, BBWV2 RNA1 NCBI accession KC790225, and RNA 2 MW556592 both from China. This is the first BBWV2 genome study reported in Australia and forms part of the strategy to integrate versatile diagnostic genomics tools at the border to safeguard the Australian grains industry.

**IMPORTANCE:**

Globally, viral diseases impair the growth and vigor of cultivated crops such as grains, leading to a significant reduction in quality, marketability, and competitiveness. As an island nation, Australia has a distinct advantage in using its border to prevent the introduction of damaging viruses, which threaten the continental agricultural sector. However, breeding programs in Australia rely on imported seeds as new sources of genetic diversity. As such, it is critical to remain vigilant in identifying new and emerging viral pathogens, by ensuring the availability of accurate genomic diagnostic tools at the grain biosecurity border. High-throughput sequencing offers game-changing opportunities in biosecurity routine testing. Genomic results are more accurate and informative compared to traditional molecular methods or biological indexing. The present work contributes to strengthening accurate phytosanitary screening, to safeguard the Australian grains industry, and expedite germplasm release to the end users.

## INTRODUCTION

Integrated diagnostic technologies such as high-throughput sequencing (HTS) offer immense opportunities in plant viruses disease management strategies. HTS approaches, such as RNA-Seq Illumina short-read sequencing, have been widely used to detect and discover RNA viruses (1 – 4). However, HTS operational cost and long turnaround times limit its full adoption for routine virus detection (5, 6). Contrary, Oxford Nanopore Technologies (ONT), such as MinIon (7), are less expensive, portable, and have timely turnaround times (8), depending on the approach selected. The ONT chemistry has rapidly progressed, making it an alternative HTS tool to identify plant pathogens successfully (9 – 12) and includes availability of a direct RNA long-read sequencing (13, 14).

The direct RNA long-read sequencing capability has proven to be robust in unravelling cell transcriptional features (15). It follows an end-to-end sequencing approach, which overcomes the complexity of transcriptome assembly required with short-read sequencing, especially in highly variable regions of a gene (16). The ONT direct RNA-Seq method directly sequences RNA without modifications in their native form and uses fewer amplification steps, which reduce PCR-associated bias, resulting in better quality sequence assemblies (13, 14, 17). Since RNA viruses are known to be notoriously variable (18), direct RNA-Seq long-read sequencing offers a faster, less complex and cost-effective sequencing approach to generate high-quality genome assemblies (19).

Broad bean wilt virus 2 (BBWV-2) belongs to the genus *Fabavirus* of the subfamily *Comovirinae,* family *Secoviridae* and is an important pathogen causing damage in many economically important horticultural and ornamental crops worldwide (20). The BBWV-2 genome has two segments of positive-sense single-stranded RNAs, RNA1 (5.8 kb) and RNA2 (3.3 kb) (20). The RNA1 encodes for a protease cofactor, a nucleoside-triphosphate (NTP)-binding motif, a VPg protease, and an RNA-dependent RNA polymerase. The RNA2 encodes for a movement protein, a large coat protein (CP), and a small CP (20). Due to its genetic variability and propensity for genome recombination (21), this virus was used to compare the Illumina RNA-Seq method with the MinION (ONT) direct-RNA-Seq method to sequence the BBWV2 genome.

## MATERIALS AND METHODS

The RNA from the symptomatic BBWV2-infected *Vicia faba* (faba bean) leaf sample “166_NSW” collected in 2001 from New South Wales, Australia, and preserved with CaCl_2_ was extracted using the RNeasy Plant Mini Kit (Qiagen), according to manufacturer’s instructions. The total RNA integrity was verified and used to prepare Illumina RNA-Seq library followed by sequencing (22). The obtained RNA-Seq (Illumina) raw reads were analyzed as previously described (22). In addition, Illumina raw reads were also merged and analyzed using CLC Genomics Workbench (version 20) (CLCGW) (CLC bio; Qiagen) ( 22). The total RNA was subjected to direct RNA sequencing using SQK-RNA002 according to manufacturer’s instructions and sequenced for 30 hours using a MinION (ONT, Oxford, UK) and a flow cell (FLO-MIN 106D R9.4.1). ONT direct RNA sequence reads were base called using fast base calling model and read filtering (min_qscore = 7) in real time using the MinKNOW software version 20.06.5 (MinKnow Core 4.1.2, Bream 6.1.4, and Guppy 4.2.2 ONT, UK). The ONT fastq raw reads were assembled using Flye v 2.9.1 (23), additional mapping in Minimap2 v 2.24 (24). Both Illumina and ONT reads contigs were subjected to BLASTN search (25), and the final consensus BBWV2 genome sequence was polished in CLCGW via long-read (beta) function. Both Illumina and ONT BBWV-2 RNA1 and RNA2 genome segments were also aligned with other BBWV2 genomes obtained from NCBI. Their alignment and phylogenetic analysis were performed using Clustal W and MEGA v 7.0.14 (26).

## RESULTS AND DISCUSSION

The Illumina RNA-Seq generated 8,687,590 reads, of which 8,669,181 reads remained after quality control. A total of 2,868,052 reads were mapped to the BBWV-2 RNA1 to yield a 5,924-nt contig that aligned to 94.07% of BBWV-2 RNA1 (KC790225) with average sequence depth of 92,061. The BBWV-2 RNA2 had a total of 3,711,210 reads mapping to it and yielded a 3,594-nt contig that aligned to 89.33% nt of BBWV-2 RNA 2 (MW556592) with a sequence depth of 73,085. The ONT direct RNA sequencing generated 1,380,000 raw reads, and 1,191,358 reads passed quality control. The ONT had a total of 75,982 reads mapping to BBWV-2 RNA1 and yielded a 5,549-nt contig that aligned to 92.6% nt of BBWV-2 RNA1 (KC790225) with a sequence depth of 2,609. The ONT BBWV-2 RNA2 had 61,847 reads mapping to it and yielded a 3,605-nt contig length that aligned to 88.0% nt of BBWV-2 RNA 2 (MW556592), with a sequence depth of 2,318.

**Fig 1 F1:**
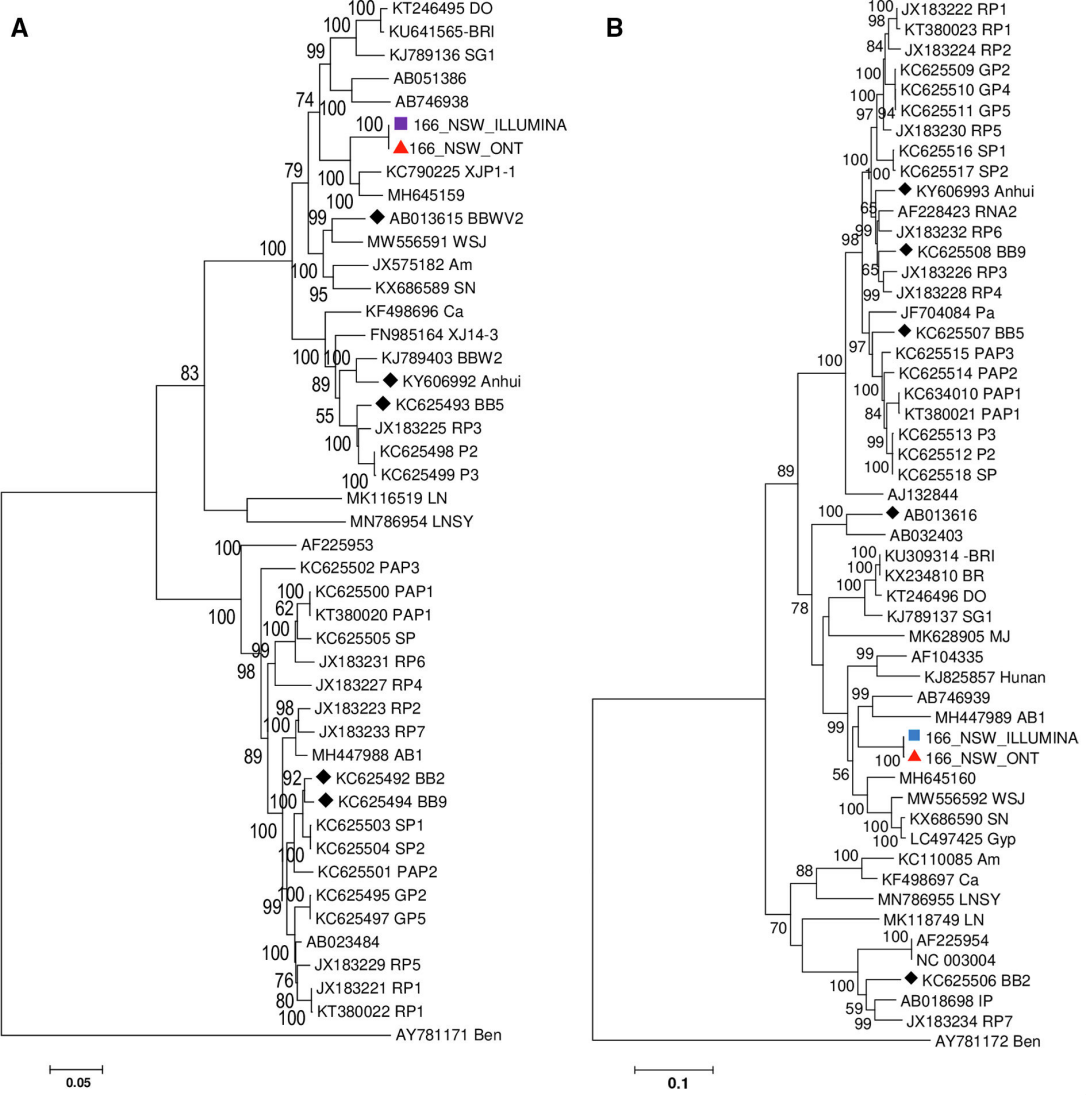
The evolutionary history was inferred using the neighbor-joining method using MEGA v7.0.14. The optimal tree with the sum of branch length = 1.73397027. The percentage of replicate trees in which the associated taxa clustered together in the bootstrap test (1,000 replicates) is shown next to the branches. The analysis involved (A) BBWV2 RNA1 45 nucleotide (nt) sequences including BBWV2 RNA1 derived from (purple square) Illumina- and (red triangle) ONT-assembled genome sequences; (B) BBBWV2 RNA2 52 nt sequences including BBWV2 RNA2 derived from (blue square) Illumina- and (red triangle) ONT-assembled genome sequences. Codon positions included were first + second + third + noncoding.

When ONT and Illumina BBWV2 RNA1 and RNA2 contigs were aligned and compared to each other, there was 98.8% and 99.1% sequence similarity for RNA1 and RNA2, respectively. The ONT and Illumina consensus sequences of BBWV2 RNA1 closely matched NCBI (KC790225) with 94.1% nt identity and BBWV2 RNA2 matched 89.3% to NCBI (MW556592). The ONT-derived 5,549 nt BBMV-2 RNA1 contig contained deletions within the NTP binding, protease factor ORFs, and the upstream of five untranslated regions (UTRs). However, the BBWV2 RNA2 3,605 nt contig generated from ONT was intact, and no sequencing errors were observed when compared to the reference sequence MW556592 and the Illumina BBWV-2 RNA2-derived genome segment. The Illumina and ONT hybrid assembled contigs for the 166_NSW BBWV-2 RNA1 and RNA2 genome segments phylogenetically grouped together and most closely aligned with the RNA 1 (KC790225) and RNA 2 (MW556592) of a none-*Fabaceae* BBWV isolate from China (Fig. 1 [Fig F1]).

**Fig 2:**
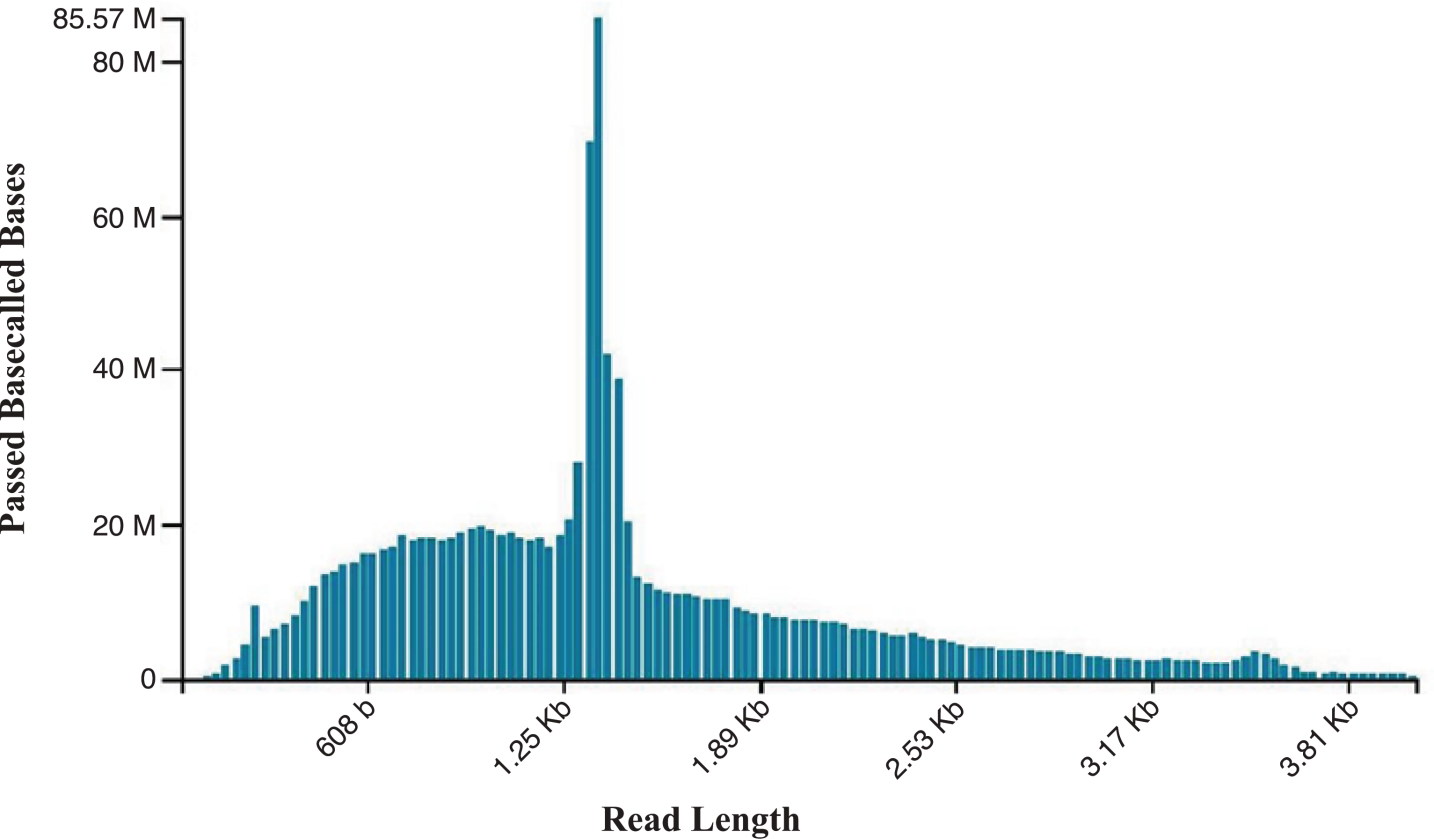
Broad bean wilt virus 2 Nanopore direct RNA long-read sequencing showing passed basecalled bases against the sequence read length.

The ONT direct RNA long-read sequencing offers an attractive option for detecting RNA viruses within a routine diagnostic framework due to native generation of long reads (Fig. 2). Also, it is cost efficient and can provide a quicker turnaround timeline (27). However, the lack of a continuous contig within RNA1 ORFs and its five UTRs that was generated by ONT may be associated with sequence error rates and a lack of plant rRNA depletion by the SQK-RNA002 sequencing kit (27). Additionally, the 22-year-old leaf material might have had its RNA degraded over time. The Illumina-derived high-quality BBWV2 RNA1 and RNA2 genome segments could be attributed to the nature of the Illumina RNA-Seq approach, which incorporated a plant ribosomal depletion step and its adaptability to low-integrity RNA. Despite the quality of ONT direct RNA sequencing of BBWV2 RNA1, being lower than Illumina sequencing, addition of rRNA followed by cDNA amplification could offer a significant cost-efficient and fast turnaround timeline option for detecting viruses routinely. As such, hybrid sequencing strategies using both long and short reads can resolve detection and genomic characterization of complex RNA viruses, benefitting from the high accuracy of Illumina and long ONT hybrid reads. Worldwide, BBWV2 has been found to be an emerging virus of economic importance due to its wide crop host range, including weeds. BBWV2 strains from different hosts have been differentiated genetically and pathogenically (28). Such studies are yet to be undertaken in Australia. The current study forms the first report of BBWV2 genome characterization in Australia and its genetic relationship with other global strains. This study reports the first BBWV-2 genome in Australia and forms part of the strategy to integrate versatile diagnostic genomics tools at the border to safeguard the Australian grains industry.

## Data Availability

The HTS reads were deposited in NCBI under BioProject PRJNA987370. The new 166_NSW genome sequence was deposited in NCBI/ENA/DDBJ under accession numbers LC772904 and LC772905.
